# Melatonin Mediates Protective Effects against Kainic Acid-Induced Neuronal Death through Safeguarding ER Stress and Mitochondrial Disturbance

**DOI:** 10.3389/fnmol.2017.00049

**Published:** 2017-02-28

**Authors:** Feixiao Xue, Cai Shi, Qingjie Chen, Weijian Hang, Liangtao Xia, Yue Wu, Sophia Z. Tao, Jie Zhou, Anbing Shi, Juan Chen

**Affiliations:** ^1^Department of Biochemistry and Molecular Biology, School of Basic Medicine and the Collaborative Innovation Center for Brain Science, Tongji Medical College, Huazhong University of Science and TechnologyWuhan, China; ^2^Department of Clinical Laboratory, Xi’an Third HospitalXi’an, China; ^3^Department of Molecular, Cellular, and Developmental Biology, University of California Santa Barbara, Santa BarbaraCA, USA; ^4^Institute for Brain Research, Huazhong University of Science and TechnologyWuhan, China; ^5^Key Laboratory of Neurological Disease of National Education Ministry, Tongji Medical College, Huazhong University of Science and TechnologyWuhan, China

**Keywords:** melatonin, kainic acid, cell death, mitochondrial dysfunction, endoplasmic reticulum stress

## Abstract

Kainic acid (KA)-induced neuronal death is linked to mitochondrial dysfunction and ER stress. Melatonin is known to protect hippocampal neurons from KA-induced apoptosis, but the exact mechanisms underlying melatonin protective effects against neuronal mitochondria disorder and ER stress remain uncertain. In this study, we investigated the sheltering roles of melatonin during KA-induced apoptosis by focusing on mitochondrial dysfunction and ER stress mediated signal pathways. KA causes mitochondrial dynamic disorder and dysfunction through calpain activation, leading to neuronal apoptosis. Ca^2+^ chelator BAPTA-AM and calpain inhibitor calpeptin can significantly restore mitochondrial morphology and function. ER stress can also be induced by KA treatment. ER stress inhibitor 4-phenylbutyric acid (PBA) attenuates ER stress-mediated apoptosis and mitochondrial disorder. It is worth noting that calpain activation was also inhibited under PBA administration. Thus, we concluded that melatonin effectively inhibits KA-induced calpain upregulation/activation and mitochondrial deterioration by alleviating Ca^2+^ overload and ER stress.

## Introduction

Neurodegeneration defines the progressive loss of function and structure of neurons that ultimately leads to neuronal death (Yildiz-Unal et al., 2015). Excitotoxicity is considered to be a major factor of neuronal death in acute and chronic neurodegenerative diseases, including Alzheimer’s disease (AD), Parkinson’s disease (PD), Huntington’s disease (HD), temporal lobe epilepsy (TLE), and amyotrophic lateral sclerosis (ALS) (Wang and Qin, 2010). Kainic acid (KA), an analog of glutamate, has been used to establish excitotoxicity models *in vitro* and *in vivo* (Sperk et al., 1983; Milatovic et al., 2001; Crespo-Biel et al., 2007; Wang et al., 2008; Li et al., 2010; Zhang and Zhu, 2011). KA associates with KA-type non-*N*-methyl D-aspartate (NMDA) receptors and causes depolarization of neurons, which can result in status epilepticus, neurodegeneration, and memory loss, etc. (Osada et al., 2010). Previous studies demonstrated that KA treatment induced neuronal apoptosis in the brain, particularly affecting neurons in the hippocampal regions (Wang et al., 2005).

The overstimulation of NMDA receptors by glutamate and its analog leads to increased levels of intracellular calcium (Ca^2+^) (Lai et al., 2014). Calcium is one of the most important signaling molecules in neurons. Maintenance of intracellular Ca^2+^ homeostasis is crucial for neuronal viability and functions (Bahar et al., 2016). Ca^2+^ influx promotes the production of reactive oxygen species (ROS), releases caspase cofactors into the cytoplasm and triggers the apoptotic cascade (Manev et al., 1989). Ca^2+^ overloading affects the mitochondrial membrane potential (MMP), which uncouples the respiratory chain and causes a decrease in ATP synthesis (Halestrap, 2009). Additionally, Ca^2+^ overloading increases the permeability of the inner mitochondrial membrane and eventually leads to mitochondrial rupture and neuronal death (Golstein and Kroemer, 2007). Mitochondria are highly dynamic organelles, undergoing continuous fission and fusion events which are critical for the maintenance of mitochondrial functions (Nasca et al., 2016). Mitochondrial fission and fusion require outer membrane fusion proteins, mitofusin 1 and 2 (Mfn-1, Mfn-2), inner membrane fusion protein optic atrophy type 1 (OPA-1), and fission protein dynamin-related protein 1 (Drp-1) (Zhao et al., 2013). Unopposed fission leads to fragmentation, whereas unopposed fusion causes over-elongation, both of which impair mitochondrial functions (Johri and Beal, 2012). In addition to mitochondrial morphology regulation, fission and fusion processes are also essential for maintaining various aspects of mitochondrial functions (Ishihara et al., 2006). The disruption mitochondrial dynamics impair mitochondrial function and cause cell death (Ishihara et al., 2006).

The endoplasmic reticulum (ER) is another vital organelle in eukaryotic cells. The ER is responsible for various cellular activities including membrane protein synthesis and maturation, lipid biogenesis, and regulation of Ca^2+^ levels (Bahar et al., 2016). ER stress is an adaptive response to restore ER homeostasis (Sano and Reed, 2013). However, prolonged ER stress will trigger apoptosis (Logue et al., 2013). Loss of cellular homeostasis induced Ca^2+^ deregulation can cause ER stress-mediated apoptosis in various pathological conditions (Kruman et al., 1998; Tombal et al., 1999; Pinton et al., 2008), which further contributes to pathophysiological conditions of neurodegenerative diseases (Lindholm et al., 2006).

As a glutamate analog, KA is able to cause excessive activation of glutamate receptors (Bleakman and Lodge, 1998). Glutamate receptor over-activation induces Ca^2+^ overloading and mitochondria functional collapse, leading to progressive neuronal death (McGeer and McGeer, 1978). KA induced Ca^2+^ influx also leads to ER membrane disintegration, ER stress and the generation of ROS, eventually leading to neuronal apoptosis and necrosis (Schinder et al., 1996; Nicholls, 2004; Kim et al., 2016). Therefore, we speculate that KA leads to neuronal apoptosis by triggering ER stress and mitochondrial dysfunction.

Melatonin (Mel), a tryptophan metabolite, is synthesized mainly by the pineal gland. Melatonin is involved in the biological regulation of circadian rhythms, sleep, moods, reproduction, tumor growth, and aging (Jean-Louis et al., 1998; Cajochen et al., 2003; Wu and Swaab, 2005; Park et al., 2010; Prieto-Domínguez et al., 2016). A previous study showed that melatonin could inhibit traumatic brain injury induced neuronal apoptosis (Mesenge et al., 1998). It has also been demonstrated that melatonin modulates Ca^2+^ levels and ROS production in a model of ischemia reperfusion stroke and helps to maintain the MMP in a model of oxygen and glucose deprivation (Hu et al., 2012; Gouriou et al., 2013; Li et al., 2014). All the evidence together suggests that the neuronal protective effects of melatonin might be associated with the modulation of intracellular Ca^2+^ levels and cellular homeostasis. Thus far, the mechanisms of how melatonin protects neurons from KA-induced apoptosis remain elusive. In this study, we show that KA affects neuron viability by Ca^2+^ overloading and mitochondrial/ER dysfunction pathways. Melatonin enacts protective effects against KA-induced neuronal apoptosis by attenuating calpain activation-induced mitochondrial dysfunction and the ER stress cascade.

## Materials and Methods

### Cell Preparation

Mouse neuroblastoma N2a cells (N2a cells) were cultured with 1:1 mixture of DMEM and Opti-MEM containing 5% fetal bovine serum (Gibco, Grand Island, NY, USA) in a humified incubator aerated with 95% air and 5% CO_2_ at 37°C. The medium was replaced every other day, and cells were plated at an appropriate density according to each experimental scale.

In experiment 1, N2a cells were treated with KA at concentrations of 0, 25, 50, and 100 μM for 8 h. 3-[4,5-dimethylthiazol-2-yl]-2,5-diphenyl-tetrazolium bromide (MTT) assay (Solarbio, Beijing, China) and crystal violet assay (Solarbio, Beijing, China) were performed to detect viability of cells. Release of lactate dehydrogenase (LDH) was detected by using LDH Cytotoxicity Detection Kit (Dojindo, Japan). To assay melatonin effects, N2a cells were pre-treated with melatonin at concentrations of 0, 25, 50, and 100 μM for 1 h before KA was added into the medium. The MTT, crystal violet and LDH assay were performed after the KA treatment.

All animal handling and surgeries were performed in accordance with the Care Standards of Laboratory Animals (China Ministry of Health Publication, 2001). Primary hippocampus neurons were prepared from 2-day-old Sprague-Dawley rats using the described protocols, with modifications (Isaev et al., 2002). The tissue was briefly digested with 0.25% trypsin in phosphate-buffered saline (PBS) for 20 min at 37°C followed by mechanical dissociation. Hippocampal neurons were seeded in poly-L-lysine-coated plates (120,000 cells/cm^2^) and grown in neurobasal medium with B-27 serum-free supplement (Gibco, Grand Island, NY, USA), 100 U/mL penicillin, 100 g/mL streptomycin, and 2 mM L-glutamine. The cultures were maintained in a humid incubator aerated with 95% air and 5% CO_2_ at 37°C. The medium was changed starting from day 4 by replacing half of the medium twice a week. Serum-free primary hippocampal cultures were utilized for the experiments after 8 days.

In experiment 2, the primary hippocampus neurons were pre-treated with or without melatonin (50 μM) for 1 h, and then stimulated with KA (50 μM) for 8 h. Melatonin (Sigma, St. Louis, MO, USA) was first dissolved in absolute ethanol at a concentration of 50 mM and diluted with culture medium to the final concentration. KA (Abcam, Cambridge, MA, USA) was first dissolved in DMSO at a concentration of 100 mM and then diluted with culture medium to the final concentration. Corresponding dilutions of ethanol and DMSO were given to the control group.

In experiment 3, the N2a cells were pre-treated with or without calpeptin (Cal, an calpain inhibitor, 20 μM, Sigma, St. Louis, MO, USA), BAPTA-AM (an Calcium chelating agent, 2.5 μM, Sigma, St. Louis, MO, USA) and sodium 4-phenylbutyrate (PBA) (Ye et al., 2014), an ER stress inhibitor, 1 mM (Sigma, St. Louis, MO, USA) for 1 h, and then treated with KA (50 μM) for 8 h.

Calpeptin and BAPTA-AM were dissolved in DMSO as stock solutions at concentrations of 20 and 5 mM, then further diluted with the cell culture medium to final concentrations of 20 and 2.5 μM. PBA was first dissolved in DMSO as stock solution at a concentration of 200 mM then diluted to 1 mM with culture medium. Corresponding dilutions of DMSO were given to the control group.

### Animals and Treatments

Adult male C57BL/6 mice, weighing 25 ± 2 g, were supplied by the Experimental Animal Center of Tongji Medical College. All experimental procedures were approved by the Animal Care and Use Committee at the Huazhong University of Science and Technology and were performed in compliance with National Institutes of Health guidelines on the ethical use of animals. The mice were housed five per cage in a room maintained at 22 ± 2°C with an alternating 12-h light–dark cycle. Food and water were available *ad libitum*.

Mice were divided randomly into four groups: KA-only group (KA), melatonin (Sigma, St. Louis, MO, USA) administration prior to KA group (Mel+KA), melatonin only group (Mel) and vehicle-treated control group (Con). Based on a previous study, mice were treated with an intraperitoneal (i.p.) injection of 30 mg/kg KA (Abcam, Cambridge, MA, USA) emulsified in 0.9% normal saline (Crespo-Biel et al., 2010). Melatonin was dissolved in absolute ethanol and diluted in saline to a final concentration of 2% ethanol before injection.

The mice in the Mel+KA group were given intraperitoneal injections of melatonin 20 mg/kg once, 30 min before the injection of KA on the first day and a single dose per day for a total of 3 days (Jain et al., 2013). As melatonin was dissolved in 2% ethanol, the control group mice received intraperitoneal injections of 2% ethanol at the same time with the same volume (0.1 mL). The mice in each group (*n* = 12) were euthanized on the 4th day after KA treatment (**Figure 2A**).

All mice were euthanized under anesthesia using 10% chloral hydrate after KA treatment, and the hippocampal tissue was harvested for further tests.

### RNA Isolation and Real-Time Polymerase Chain Reaction Quantification

Total RNA was isolated from the N2a cells by using RNase Mini Kit (Qiagen, Valencia, CA, USA) following the manufacturer’s instructions. Primer sequences (Greene et al., 2015) were listed in **Table 1**.

**Table 1:** Primer sequences for real-time PCR.

Protein types	Primer sequence
OPA-1	Forward: 5′-TCT GAG GCC CTT CTC TTG TT-3′ Reverse: 5′-TCT GAC ACC TTC CTG TAA TGC T-3′
Drp-1	Forward: 5′-TCA CCC GGA GAC CTC TCA TT-3′ Reverse: 5′-TGCTTCAACTCCATTTTCTTCTCC-3′
Mfn-1	Forward: 5′-TTG CCA CAA GCT GTG TTC GG-3′ Reverse: 5′-TCT AGG GAC CTG AAA GAT GGG C-3′
Mfn-2	Forward: 5′-AGA GGC AGT TTG AGG AGT GC-3′ Reverse: 5′-ATG ATG AGA CGA ACG GCC TC-3′
VDAC-1	Forward : 5′-CCT CCC ACA TAC GCC GAT CT-3′ Reverse: 5′-TTA AGC CAA AGC CGT AGC CC-3′
GAPDH	Forward : 5′-ACG GAT TTG GTC GTA TTGGG-3′ Reverse: 5′-CGC TCC TGG AAG ATG GTGAT-3′

All of the primers were synthesized by Sangon Biotech (Shanghai, China). Real-time polymerase chain reaction (PCR) for cDNA analysis was conducted at 60–95°C for 45 cycles on a Sequence Detection System (ABI Prism 7000, Applied Biosystems, Darmstadt, Germany) following the manufacturer’s instructions and using SYBR Green Reaction Master Mix (TaKaRa, Dalian, China). For each sample, VDAC-1 or GAPDH served as the housekeeping gene. Fold-change expression was calculated from the threshold cycle (Ct) values. For calculation of relative changes, gene expression measured in control tissues was taken as the baseline value.

### Western Blotting

Total proteins were extracted by using a protein extraction kit (Pierce, IL, USA) in accordance with the manufacturer’s instructions. Protein extracts were dissolved in 15% sodium dodecyl sulfate polyacrylamide gel, and then transferred to a nitrocellulose membrane at 150 mA. After being blocked with 5% non-fat skim milk [diluted with Tris-buffered saline containing 0.1% Tween 20 (TBST)] for 1 h at room temperature, the membrane containing the protein extracts was incubated overnight with primary antibody (diluted with 2% bovine serum albumin in TBST) at 4°C. The following primary antibodies were used: anti-GAPDH (1:3000, Abcam, Santa Cruz, CA, USA); anti-GRP78 (1:2000, Abcam, Cambridge, MA, USA); anti-CHOP (1:500, Abcam, Cambridge, MA, USA); anti-Mfn-1 (1:1000, Abcam, Cambridge, MA, USA), anti-Mfn-2 (1:1000, Abcam, Cambridge, MA, USA), anti-OPA-1 (1:1000, CST, Danvers, MA, USA), anti-Drp-1 (1:1000, Santa Cruz, CA, USA), anti-calpain (1:1000, Santa Cruz, CA, USA); anti-Cyt C (1:1000, Santa Cruz, CA, USA); anti-cleaved caspase-12 (1:1000, CST, Danvers, MA, USA) and anti-cleaved caspase-3 (1:1000, CST, Danvers, MA, USA), anti-cleaved caspase-9 (1:1000, CST, Danvers, MA, USA) and anti-VDAC-1(1:1500, Abcam, Cambridge, MA, USA). On the second day, proteins were visualized using the enhanced chemiluminescence detection system (Pierce, IL, USA) after incubating with respective horseradish peroxidase-conjugated secondary antibodies (1:1000, Amersham Pharmacia Biotech, Buckinghamshire, UK) and then exposed to medical x-ray film. The intensity of the blots was quantified using a gel-image analyzer (JS380; Peiqing Science and Technology, Shanghai, China).

### Calpain Activity Assay

The calpain activity was measured using Calpain Substrate II Kit (Merck Millipore, Darmstadt, Germany). Cells were washed twice with PBS and lysed on ice with extraction buffer. The total protein extracted (50 μg) was incubated with 150 μL substrate and 1 mL reaction buffer for 100 min at 37°C. The release of 7-amino-4-methyl-coumarin (AMC) from the reaction was monitored at an emission of 440 nm using a fluorescence spectrometer. Fluorescence units were converted into AMC release using the standard curve. Activity of calpain was expressed and documented in pmol of AMC cleaved per minute per milligram of protein.

### Time-Lapse Imaging and Mitochondrial Morphology Analyses

To observe the changes in the mitochondrial morphology in N2a cells, the KA and/or melatonin-treated N2a cells were incubated with the MitoTracker Red CMXRos probe (250 nM) (Invitrogen, Carlsbad, CA, USA) for 30 min at 37°C. After being washed three times in cold PBS, the cells were visualized under a Nikon C2 confocal laser scanning microscope (Nikon, Tokyo, Japan) with excitation of 579 nm and emission greater than 599 nm. For morphological quantification in neurites, z-sections were merged (using maximal projection) and the entire length (from tip to tip) of MitoTracker Red labeled mitochondria of neurites was measured. In cell bodies, mitochondria length was measured in each z-section of the entire soma. Quantification of mitochondria length was performed by using ImageJ software as previously described (De Vos and Sheetz, 2007). The number of mitochondria was counted in control N2a cells (*n* = 50) and experimental groups (*n* = 40). Statistical significance was determined using one-way ANOVA analysis.

### JC-1 Dye Membrane Potential Staining

Mitochondrial membrane potential in N2a cells was measured by using MMP assay kit with JC-1 (Beyotime Biotechnology, Shanghai, China). All procedures followed the manufacture’s instructions. The fluorescence intensities were measured using a fluorescence plate reader at 590 nm (red) and 529 nm (green). According to the ratio of fluorescence intensities at 590 and 529 nm, the loss of MMP was assessed and recorded.

### TUNEL Assay

Apoptosis was assessed by detecting DNA fragmentation through TUNEL staining. TUNEL staining for apoptotic cells were performed in accordance the manufacturer’s instructions (Roche Corporation, Germany). The N2a cells growing on the cover glass slide were fixed. Endogenous peroxidase activity was inhibited by immersing the sections in methanol with 0.3% H_2_O_2_ for 20 min at room temperature. After washing with distilled water and 0.01 M PBS, the slides were soaked in TdT (terminal deoxynucleotidyl transferase) buffer at room temperature for 15 min and then incubated with TdT buffer containing 5 U TdT enzyme and 0.5 nmol biotinylated 16-dUTP in a humidified chamber for 1 h at 37°C. The reaction was terminated by adding 2× sodium saline citrate (SSC). The sections were then incubated with HRP-conjugated streptavidin according to the manufacturer’s instructions. Colorimetric development of the reaction was done by incubation in DAB (diaminobenzidine) solution for 5 min. For the negative control slides, either TdT enzyme or biotinylated 16-dUTP was omitted in the labeling reaction. The specimens in all groups (*n* = 3) were observed using an upright microscope (OLYMPUS BX53F, Japan) and in at least six random fields of high-power (×100).

### ROS Determination

N2a cells were labeled with DCFH-DA (Sigma, St. Louis, MO, USA) (10 μM) for 30 min after KA treatment (Liu et al., 2015). ROS generation was indicated by green florescence and visualized using a fluorescence microscope (Olympus, Japan). The fluorescence in each group was assessed by flow cytometry with an excitation wavelength of 488 nm and an emission wavelength of 525 nm.

### Transmission Electron Microscope

To determine the effects of KA and melatonin on the morphology of mitochondria, we conducted transmission electron microscopy (TEM) of N2a cells from control and experimental treatments (*n* = 4). N2a cells were fixed in 2.5% glutaraldehyde and post-fixed in 2% OsO_4_ at room temperature. Cellular staining was performed at 4°C for 2 h in 2% uranyl acetate in the dark. Samples were rinsed in sodium phosphate buffer (0.1 M, pH 7.2), dehydrated in ethanol and infiltrated overnight in Araldite. Following polymerization, embedded samples were detached from the chamber slide and glued to Araldite blocks. Serial semi-thin (1.5 mm) sections were mounted onto slides and stained with 1% toluidine blue. The selected semi-thin sections were glued (Super Glue, Loctite) to araldite blocks and detached from the glass slide. Ultrathin (0.07 mm) sections were prepared and stained with lead citrate. Finally, photomicrographs were obtained under a TEM using a digital camera (Hitachi, Japan).

### Intracellular Ca^2+^ Measurement

Intracellular Ca^2+^ ([Ca^2+^]_i_) was measured as previously described (Blatter and Wier, 1990). To measure the acute effect of KA on [Ca^2+^]i change, N2a cells were grown on cover slides and washed three times with 2 μM Fura-2 acetoxymethyl ester (Fura-2 AM) in Hanks Balanced Salt Solution (HBSS, containing 145 mM NaCl, 5 mM KCl, 1 mM MgCl_2_, 2 mM CaCl_2_, 0.75 mM Na_2_HPO_4_, 10 mM glucose, and 10 mM HEPES, pH 7.4). N2a cells were then incubated in the same solution for 30 min at 37°C. In calcium-free experiments, EGTA (100 μM) was substituted by CaCl_2._

Before each experiment, the cover slides were mounted on a chamber positioned on the movable stage of an inverted Olympus IX-70 microscope equipped with a calcium imaging system (TILL Photonics Gmbh, Germany) and super fused by HBSS for 10 min. Fura-2 AM loaded cells were illuminated at 340 nm for 150 ms and 380 nm for 50 ms at 1 s intervals using a TILL polychrome monochromator. Fura-2 fluorescence emission was imaged at 510 nm by a cooled-CCD (TILL Photonics Gmbh, Germany) through a X-70 Fluor oil immersion lens (Olympus, Tokyo, Japan) and a 460 nm long pass barrier filter. F340/F380 fluorescence ratios were generated by Olympus software. Paired F340/F380 fluorescence ratio images were acquired every 3 s for [Ca^2+^]_i_. In the experiment, the ratio between F380 of Ca^2+^ free solutions and F380 of high calcium medium was recorded as coefficient factor β. Intracellular Ca^2+^ concentration ([Ca^2+^]_i_) = Kd × β × [(Ratio-R_min_)/(R_max_-Ratio)], a constant Kd is the dissociation constant of Fura-2 AM for Ca^2+^ and was assumed to be 224 at 37°C. R_max_ is the ratio obtained after ionomycin treatment. R_min_ is the ratio obtained from Ca^2+^ free solution.

Data was obtained by evaluating the fluorescence immediately after KA stimulation with subtraction of background fluorescence and division by the fluorescence intensity before KA stimulation. For the melatonin treatment groups, melatonin (50 μM) was added to dishes 1 h before KA stimulation.

### ATP Measurement

The concentration of ATP was determined by using ATP determination kit (Thermo Fisher Scientific, Inc., Waltham, MA, USA). All procedures followed the manufacture’s instructions. Samples were incubated with ATP reaction mixture for 30 min and detected at 560 nm. A standard curve was established to calculate the concentration of samples.

### Neuronal Loss Assay

Hippocampal tissues were embedded and sectioned at 10 μm, with three independent samples for each group. After staining with 1% toluidine blue for 15 min, the sections were dehydrated and mounted for microscopic examination. CA3 areas of the hippocampus were examined with an Olympus AX-70 microscope equipped with a motorized stage (Olympus, Astoria, NY, USA). Images were captured at 20× magnification in every section. For each independent sample, surviving neurons exhibiting normal morphology with positive blue staining Nissl bodies were included in counts.

### Statistical Analysis

Data was expressed as mean ± standard deviation and analyzed using SPSS 10.0 statistical software (SPSS, Inc., Chicago, IL, USA). The one-way analysis of variance followed by least significant difference *post hoc* test was used to determine the significance of differences among groups (*P* < 0.05, *P* < 0.01, and *P* < 0.001).

## Results

### Melatonin Remarkably Mitigates KA-Induced Apoptosis

**FIGURE 1:**
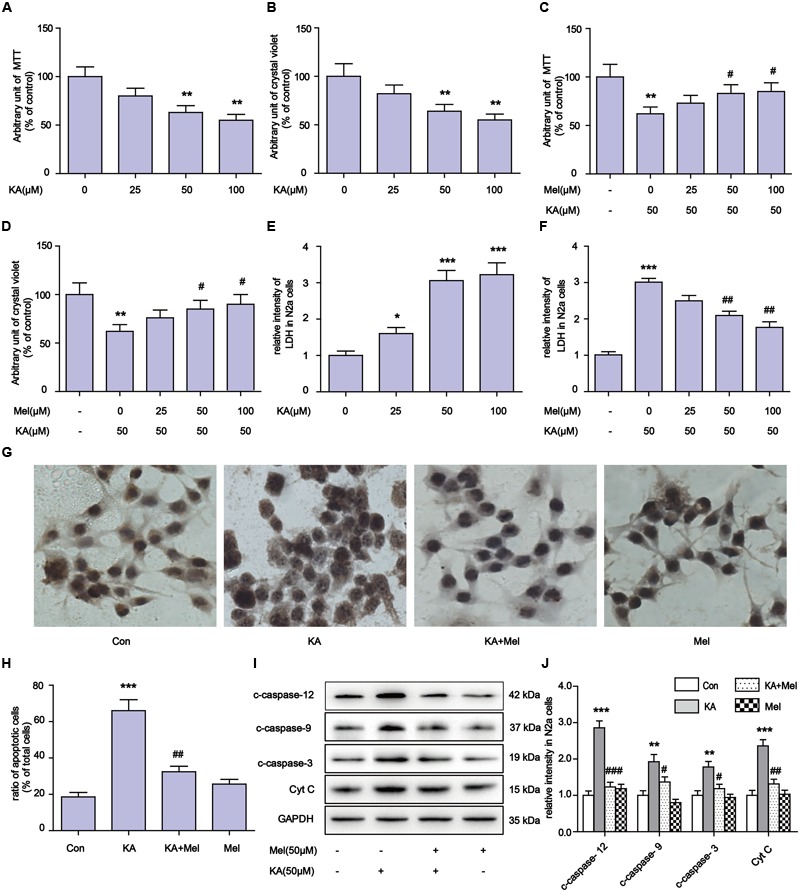
**Melatonin mitigates kainic acid (KA)-induced N2a cells apoptosis. (A)** N2a cells viability were assayed by MTT after treatment with 0, 25, 50, 100 μM KA. **(B)** N2a cells viability were assayed by crystal violet after treatment with 0, 25, 50, 100 μM KA. **(C)** N2a cells viability were assayed by MTT after treatment with both melatonin and KA. **(D)** N2a cells viability were assayed by crystal violet after treatment with both melatonin and KA. **(E)** LDH release after treatment with 0, 25, 50, 100 μM KA. **(F)** LDH release after treatment with both melatonin and KA. **(G,H)** N2a cells apoptosis was assayed by tunnel staining. **(I)** The levels of c-caspase-12, -9, -3 and Cyt C in N2a cells. **(J)** Expression analysis of c-caspase-12, -9, -3 and Cyt C (*^∗^P* < 0.05, *^∗∗^P* < 0.01, *^∗∗∗^P* < 0.001 vs. controls; *^#^P* < 0.05, *^##^P* < 0.01, *^###^P* < 0.001 vs. the KA group; significant difference from the respective values determined by one-way analysis of variance test. *n* = 3 for western blots and tunnel assay).

Kainic acid is an analog of glutamate with the capability of associating with specific KA-type non-NMDA receptors. KA has been reported to induce status epilepticus, neurodegeneration and memory loss (Sperk et al., 1983). To validate the neuronal toxicity of KA, we treated N2a cells with KA at a series of concentrations (0, 25, 50, 100 μM). In MTT and crystal violet assays, KA decreased cell metabolism and cell viability in a dose-dependent manner (**Figures 1A–D**). KA application elicited the activation of caspases and the release of cytochrome C (Cyt C), which are the characteristic phenotypes of apoptosis (**Figures 1I,J**). In the assays, we noticed that application of 50 or 100 μM of KA induced significant apoptosis and toxicity. There were no detectable differences between these doses on cell viability and the release of LDH (**Figures 1E,F**).

To determine whether melatonin could inhibit the apoptosis of N2a cells, we added different concentrations (50 and 100 μM) of melatonin into cell cultures. As shown in **Figures 1I,J**, melatonin addition greatly decreased the expression of c-caspase-3 (cleaved caspase-3), c-caspase-12, c-caspase-9, and Cyt C. The inhibitory effect of melatonin on cell apoptosis was also assessed by TUNEL assay (**Figures 1G,H**). We observed that 50 μM melatonin was enough to regain cell viability and reduce LDH release effectively (**Figures 1C,D,F**). The cell viability and LDH release results between application of 50 and 100 μM melatonin were not significantly different.

**FIGURE 2:**
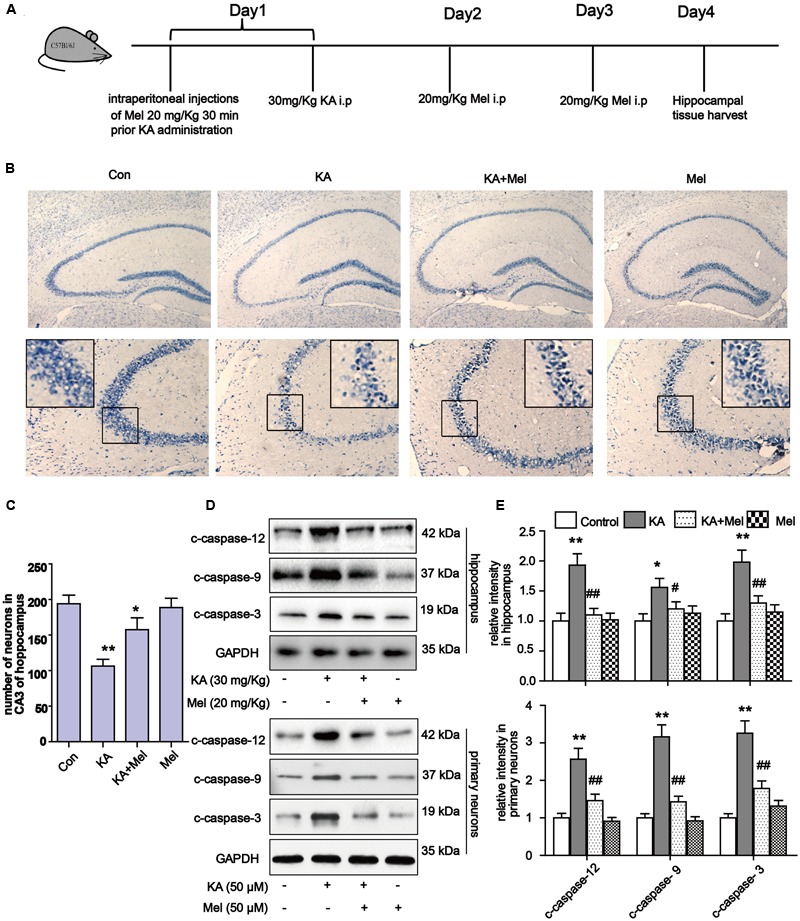
**Melatonin reduces KA-induced neuron loss *in vivo.* (A)** The administration procedure of melatonin and KA in C57 BL/6 mice. **(B)** Nissil’s staining assay was used to examine neuronal loss in KA and/or melatonin treated animals hippocampus tissue. Neuron numbers in hippocampus CA3 area were quantified in **(C)**. **(D,E)** Expression levels of c-caspase-12, -9, -3 in hippocampal tissues and primary neurons. ^∗^*P* < 0.05, ^∗∗^*P* < 0.01, ^∗∗∗^*P* < 0.001 vs. controls; ^#^*P* < 0.05, ^##^*P* < 0.01, ^###^*P* < 0.001 vs. the KA group; significant difference from the respective values determined by one-way analysis of variance test. *n* = 7 for Nissil’s staining; *n* = 3 for western blots.

To determine whether KA affects neuronal viability *in vivo*, we analyzed neuronal loss in KA and/or melatonin treated C57BL/6 mice. Adult male C57BL/6 mice were treated with an intraperitoneal (i.p.) injection of 30 mg/kg KA. The mice in the Mel+KA group were given intraperitoneal injections of 20 mg/kg melatonin 30 min before KA injection, and an additional dose on day 2 and day 3 (**Figure 2A**). Hippocampal tissues were stained with toluidine blue. We examined CA3 areas of the hippocampus and counted the surviving neurons with blue Nissl bodies (**Figure 2B**). Melatonin application greatly increased the density of surviving neurons (**Figure 2C**), indicating that melatonin functions autonomously in the hippocampus to promote the survival of neurons. Next, we assayed the expression of c-caspase-12, -9, -3 in hippocampal tissue and primary neurons. Similar to the N2a cells results, quantitative analysis of expression levels of c-caspase-12, -9, -3 in both hippocampal tissue and primary neurons showed that melatonin significantly reduced the levels of c-caspase-3, c-caspase-12, and c-caspase-9 *in vivo* (**Figures 2D,E**).

### Melatonin Ameliorates KA-Induced Mitochondrial Fragmentation and Dysfunction

**FIGURE 3:**
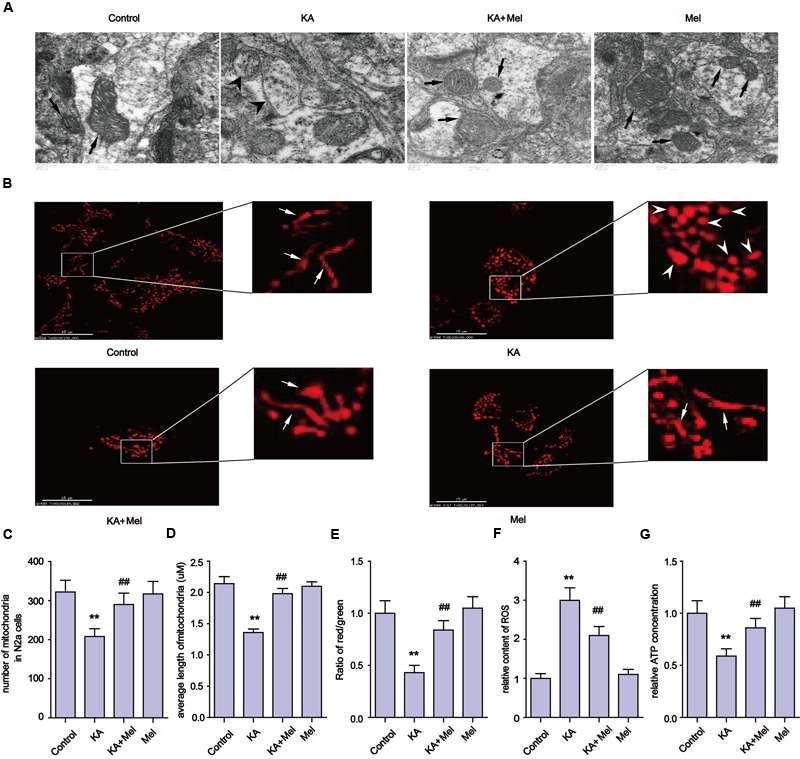
**Melatonin ameliorates KA-induced mitochondrial fragmentation and dysfunction. (A**) Transmission electron microscope images of mitochondrial ultrastructure in KA and/or melatonin-treated N2a cells. **(B)** The morphology of mitochondria stained by mito-tracker in KA and/or melatonin-treated N2a cells. **(C)** Numbers of mitochondria in KA and/or melatonin-treated N2a cells. **(D**) Average length of mitochondria in KA and/or melatonin-treated N2a cells. **(E)** Mitochondrial membrane potential (MMP) in KA and/or melatonin-treated N2a cells. **(F)** ROS production in KA and/or melatonin-treated N2a cells. **(G)** ATP concentration in KA and/or melatonin-treated N2a cells (*^∗∗^P* < 0.01 vs. controls; *^##^P* < 0.01 vs. the KA group; significant difference from the respective values determined by one-way analysis of variance test. *n* = 3).

Mitochondria defects are closely associated with apoptosis (Chen et al., 2007). To examine the integrity of mitochondria in KA-treated N2a cells, we first used TEM to assay the ultrastructure of mitochondria in N2a cells (**Figure 3A**). We observed that mitochondria in the KA-treated group presented swelling, crest fracture, and disappearance (**Figure 3A**). Next, we also assessed the mitochondrial morphology changes. We found that the mitochondria displayed canonical tubular patterns under physiological conditions (control group), while KA exposed mitochondria turned into puncta-like fragments (**Figure 3B**). The average length of mitochondria was shorter after KA treatment compared to that of the control group mitochondria (**Figure 3D**). These observations indicate the occurrence of mitochondrial fragmentation. Also, we assayed the morphology of mitochondria in KA and/or melatonin-treated rat primary neurons. Consistently, melatonin ameliorates KA-induced mitochondrial fragmentation. The number and the average length of mitochondria in both KA and melatonin-treated group were significantly restored compared to that of the mitochondria in the KA-only group (**Supplementary Figures S1A–C**).

In addition to the morphological impairments, we also observed a decrease in the average number of mitochondria per cell in the KA-treated group (**Figure 3C**). To examine if KA affects mitochondrial functions, we assayed the levels of ROS, ATP and the MMP in N2a cells. As shown in **Figures 3E–G**, KA treatment caused ROS accumulation, MMP collapse and a decrease in ATP production, indicating the loss of mitochondrial functions.

In an effort to characterize the protective effects of melatonin on mitochondria, we examined the subcellular pattern of mitochondria in both KA and melatonin-treated groups. Melatonin application significantly ameliorated KA-induced mitochondria morphological changes and ROS level spikes. In addition, melatonin restored ATP production and MMP in both KA and melatonin-treated groups. These observations indicate that melatonin is an effective inhibitor of KA-induced mitochondrial fragmentation and dysfunction.

### Melatonin Relieves KA-Induced Mfn-2 Degradation

As highly dynamic organelles, mitochondria undergo continuous fission and fusion events (Nicholls, 2004). Unopposed fission leads to fragmentation, whereas unopposed fusion results in abnormal elongation, both of which would greatly impair mitochondrial functions (Johri and Beal, 2012). Mitochondrial fission and fusion are tightly regulated by mitochondrial fission and fusion proteins. Drp-1 is required for fission (Shen et al., 2014), while Mfn-1, Mfn-2, and OPA-1 are critical for fusion (Chen and Chan, 2009). In addition to morphology maintenance, fission and fusion processes are essential for various aspects of mitochondrial function (Ishihara et al., 2006). The disruption of mitochondrial dynamics causes its morphological and functional impairments (Ishihara et al., 2006).

**FIGURE 4:**
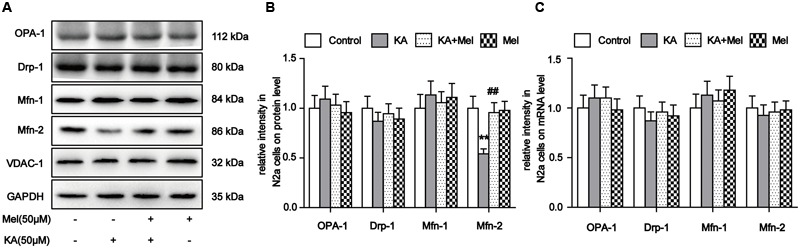
**Melatonin relieves KA-induced Mfn-2 degradation. (A)** Expression of OPA-1, Drp-1, Mfn-1, and Mfn-2 in KA and/or melatonin-treated N2a cells. **(B)** Relative protein level of OPA-1, Drp-1, Mfn-1, and Mfn-2 in KA and/or melatonin-treated N2a cells. **(C**) Relative mRNA level of OPA1, Drp-1, Mfn-1, and Mfn-2 in KA and/or melatonin-treated N2a cells (*^∗∗^P* < 0.01 vs. controls; *^##^P* < 0.01 vs. the KA group; significant difference from the respective values determined by one-way analysis of variance test. *n* = 3).

Our evidence suggests that mitochondrial morphology and functions can be affected by KA treatment. To determine whether KA perturbs mitochondrial dynamic regulators’ homeostasis, we examined the expression levels of proteins involved in mitochondrial fusion and fission. The protein level of Mfn-2 decreased significantly after KA treatment, while the levels of Drp-1, OPA-1, and Mfn-1 remained unchanged (**Figures 4A,B**). Next, we examined the mRNA levels of these factors. Surprisingly, the mRNA levels of all proteins remained unaffected (**Figure 4C**). As suggested by previous study (Wang et al., 2015), KA probably decreased the protein levels of Mfn-2 by prompting protein degradation but not by blocking expression. Furthermore, application of melatonin reversed the protein levels of Mfn-2 in N2a cells, which is consistent with the protective effects of melatonin on mitochondrial morphology and functions (**Figures 4A,B**). Therefore, we speculate that the inhibitory role of melatonin on mitochondrial dysfunction could be the alleviation of mitochondrial dynamic defects.

### Melatonin Alleviates KA-Induced Increased Ca^2+^ Levels and Calpain Activation

**FIGURE 5:**
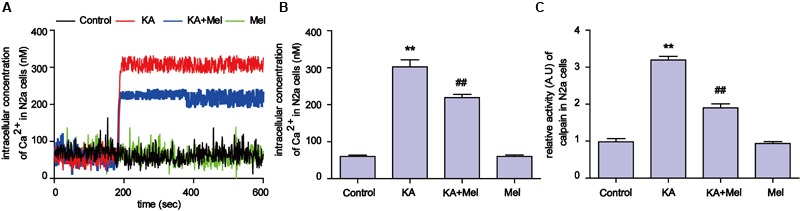
**Melatonin alleviates KA-induced Ca^2+^ elevation and calpain activation. (A)** Fura-2 AM probe was used to determine real-time Ca^2+^ concentration in KA and/or melatonin-treated N2a cells. **(B**) Concentration of total intracellular Ca^2+^ in KA and/or melatonin-treated N2a cells. **(C)** Relative activity of calpain in KA and/or melatonin-treated N2a cells (*^∗∗^P* < 0.01 vs. controls; *^##^P* < 0.01 vs. the KA group; significant difference from the respective values determined by one-way analysis of variance test. *n* = 3 for Ca^2+^ image; *n* = 5 for activity assay).

Ca^2+^ homeostasis disorder was observed in a variety of diseases (Wrogemann and Pena, 1976). Upon overstimulation of NMDA receptors, Ca^2+^ accumulates abnormally and impairs mitochondrial function, leading to a decrease in ATP production and an increased release of ROS (Halestrap, 2009). To try to understand the molecular mechanism underlying mitochondrial defect relief by melatonin treatment, we assayed intracellular Ca^2+^ concentration and calpain activity. As shown in **Figures 5A,B**, after KA treatment, intracellular Ca^2+^ was remarkably increased compared to the control group (∼6 fold increase). In contrast, melatonin treatment effectively decreased the levels of intracellular Ca^2+^ in KA and melatonin-treated N2a cells (**Figures 5A,B** and **Supplementary Figures S2A,B**). Due to the Ca^2+^ influx, the activity of calpain increased ∼3 fold in the KA-treated group. Melatonin treatment significantly decreased the abnormally elevated activity of calpain (**Figure 5C**). Together with the inhibitory effect of melatonin on Ca^2+^ influx, our results suggest that melatonin could alleviate KA induced mitochondrial dysfunction and apoptosis through attenuating increased Ca^2+^ levels and calpain activation.

### Blocking KA-Induced Ca^2+^ Increase and Calpain Activation Can Effectively Relieve Mitochondrial Defects

**FIGURE 6:**
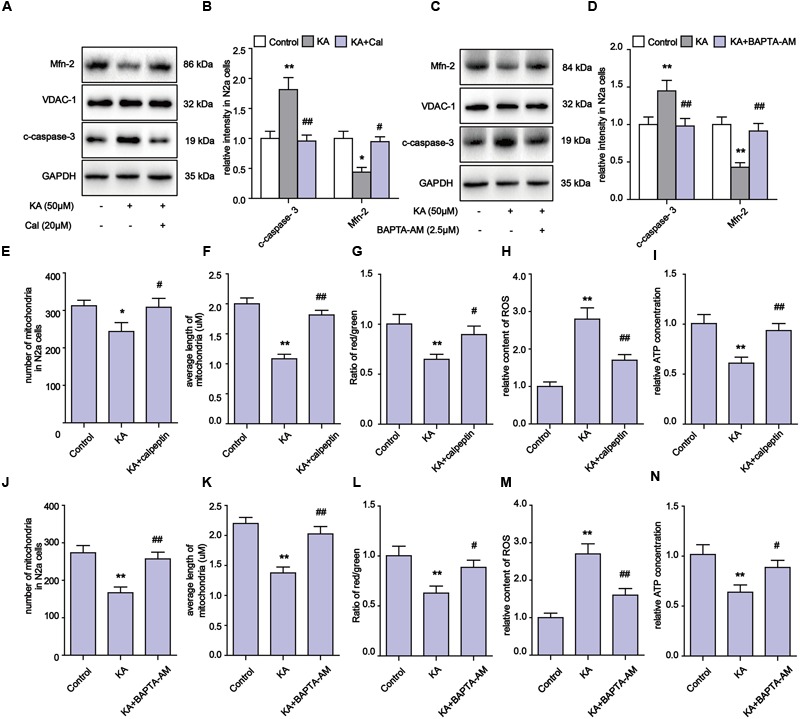
**Blocking KA-induced Ca^2+^ elevation and calpain activation can effectively relieve mitochondrial defects. (A,B)** Expression levels of Mfn-2 and c-caspase-3 in KA, KA+calpeptin treated N2a cells. **(C,D)** Expression level of Mfn-2 and c-caspase-3 in KA, KA+BAPTA-AM treated N2a cells. **(E)** Number of mitochondria in KA, KA+calpeptin treated N2a cells. **(F)** Average length of mitochondria in KA, KA+calpeptin treated N2a cells. **(G)** MMP was assayed by ratio of red/green fluorescence intensity in KA, KA+calpeptin treated N2a cells. **(H)** Relative production of ROS in KA, KA+calpeptin treated N2a cells. **(I)** Relative production of ATP in KA, KA+calpeptin treated N2a cells. **(J**) Mitochondria number in KA, KA+BAPTA-AM treated N2a cells. **(K)** Average length of mitochondria in KA, KA+BAPTA-AM treated N2a cells. **(L)** MMP in KA, KA+BAPTA-AM treated N2a cells. **(M)** Relative production of ROS in KA, KA+BAPTA-AM treated N2a cells. **(N)** Relative production of ATP in KA, KA+BAPTA-AM treated N2a cells (*^∗^P* < 0.05, *^∗∗^P* < 0.01 vs. controls; *^#^P* < 0.05, *^##^P* < 0.01 vs. the KA group; significant difference from the respective values determined by one-way analysis of variance test. *n* = 3).

Our evidence suggests that the inhibitory effects on Ca^2+^ overloading and calpain activation by melatonin could account for the alleviation of KA-induced mitochondrial dysfunction. To determine if the mitochondrial impairments were due to Ca^2+^ overloading and calpain activation, we used calpeptin and BAPTA-AM to inhibit calpain activity and Ca^2+^ overloading, then examined the levels of Mfn-2 and caspase-3. We observed that both calpeptin and BAPTA-AM fully reversed the decrease in Mfn-2 and increase in cleaved caspase-3 in KA-treated cells (**Figures 6A–D**). Furthermore, applications of either calpeptin or BAPTA-AM led to significant improvements in mitochondrial functions and morphology (**Figures 6E–N**). In addition, calpeptin or BAPTA-AM treatments decreased ROS production, restored ATP and MMP levels, and improved the number and morphology of mitochondria. Together, the data indicates that the KA-induced mitochondrial defects were due to increased Ca^2+^ levels and calpain activation. Melatonin treatment can greatly restore mitochondrial dynamics by inhibiting aberrant Ca^2+^ levels and calpain activation.

### KA-Induced ER Stress Contributes to Mitochondrial Defects and Apoptosis and Melatonin Can Effectively Suppress ER Stress

**FIGURE 7:**
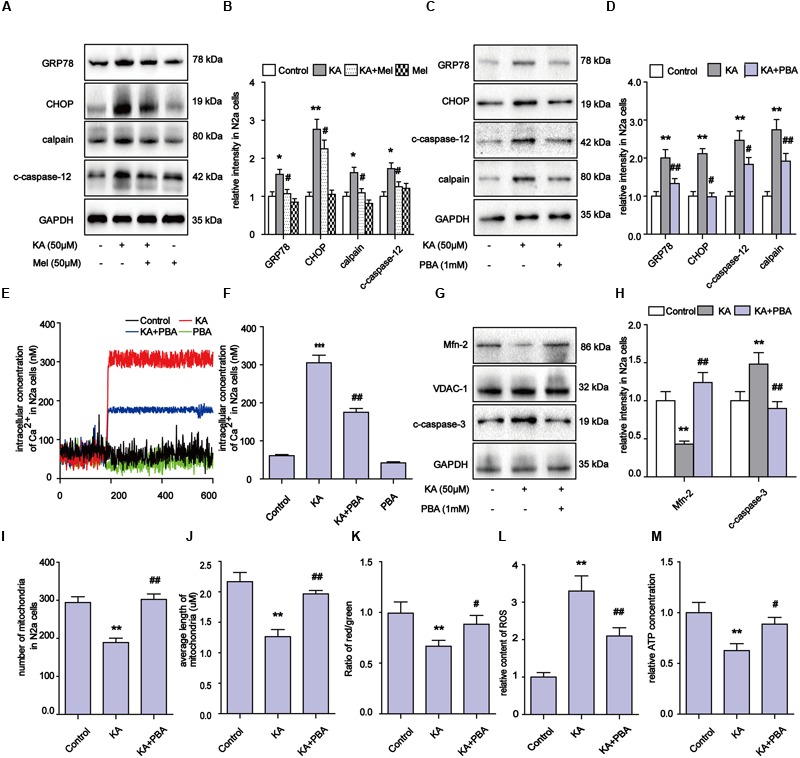
**Kainic acid-induced ER stress contributes to mitochondrial defects and apoptosis and melatonin can effectively suppress ER stress. (A,B)** Expression levels of GRP78, CHOP, calpain, c-caspase-12 in KA and/or melatonin treated N2a cells. **(C,D)** Expression levels of GRP78, CHOP, calpain, c-caspase-12 in KA and/or PBA treated N2a cells. **(E,F**) Fura-2 AM probe was used to measure real-time Ca^2+^ concentration in KA and/or PBA treated N2a cells. **(G,H)** Expression levels of Mfn-2 and c-caspase-3 in KA, KA+PBA treated N2a cells. **(I)** Mitochondria number in KA, KA+PBA treated N2a cells. **(J)** Average length of mitochondria in KA, KA+PBA treated N2a cells. **(K)** MMP in KA, KA+PBA treated N2a cells. **(L)** Relative production of ROS in KA, KA+PBA treated N2a cells. **(M)** Relative content of ATP in KA, KA+PBA treated N2a cells (*^∗^P* < 0.05, *^∗∗^P* < 0.01, *^∗∗∗^P* < 0.001 vs. controls; *^#^P* < 0.05, *^##^P* < 0.01 vs. the KA group; significant difference from the respective values determined by one-way analysis of variance test. *n* = 3).

Upon activation by intracellular Ca^2+^ overloading, calpain participates in a series of physiological and pathological processes, including ER stress (Wu and Lynch, 2006). Specifically, calpain activates the expression of GRP78, CHOP, and eventually triggers apoptosis regulators including caspase-12 and caspase-3 (Chakraborti et al., 2012). After KA treatment, the levels of calpain, GRP78, CHOP and c-caspase-12 significantly increased (**Figures 7A–D**). These observations suggest that the apoptosis after KA treatment could be partially due to ER stress. Further analyses show that melatonin or PBA application effectively downregulated the increased levels of Ca^2+^ and ER stress related proteins in the KA group (**Figures 7A–F**).

Thus far, our results suggest that melatonin could inhibit apoptosis through regulating ER stress as well. To determine if ER stress also participates in the modulation of mitochondrial dynamics, we examined the levels of Mfn-2 and caspase-3, ROS production, MMP, ATP production and mitochondria morphology in N2a cells (**Figures 7G–M**). PBA administration greatly alleviated the KA-induced degradation of Mfn-2, indicating that inhibiting ER stress contributes to the protective effects of melatonin on mitochondrial dynamics (**Figures 7G,H**). Also, the decreased ROS levels and improved ATP production/MMP/mitochondria morphology suggest that KA-induced ER stress contributes to mitochondrial dysfunction and apoptosis, and that melatonin can improve mitochondrial functions via ER stress inhibition (**Figures 7I–M**).

**FIGURE 8:**
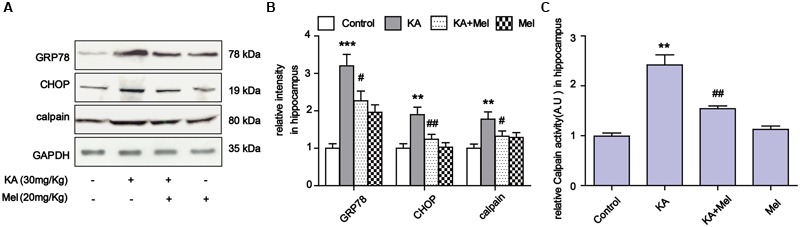
**Melatonin suppressed KA-induced ER stress *in vivo.* (A)** Expression of GRP78, CHOP and calpain in KA and/or melatonin-treated animals hippocampus. **(B**) Relative analysis of the expression levels of GRP78, CHOP and calpain in KA and/or melatonin-treated animals hippocampus. **(C)** Relative activity of calpain in KA and/or melatonin-treated animals hippocampus. ^∗∗^*P* < 0.01, ^∗∗∗^*P* < 0.001 vs. controls; ^#^*P* < 0.05, ^##^*P* < 0.01 vs. the KA group; significant difference from the respective values determined by one-way analysis of variance test. *n* = 3 for western blots; *n* = 5 for calpain activity assay.

To examine the ER stress alleviation effects of melatonin *in vivo*, we assayed the expression levels of GRP78, CHOP and calpain in KA and/or melatonin-treated C57BL/6 mice. Consistent with the results in N2a cells, KA induced GRP78, CHOP and calpain overexpression were effectively reduced *in vivo* (**Figures 8A,B**). Increased calpain activity was also suppressed after melatonin application (**Figure 8C**).

## Discussion

**FIGURE 9:**
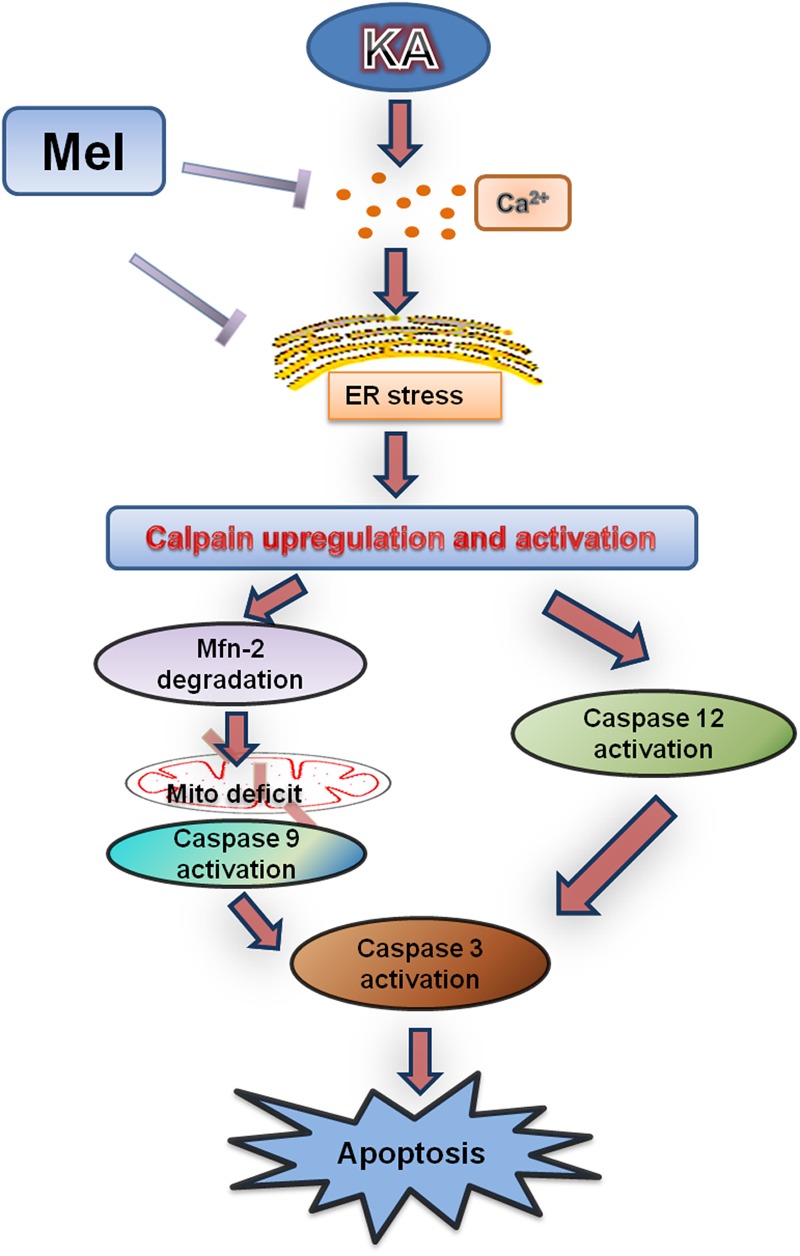
**Functional model of melatonin protecting effects against KA-induced neuronal death.** KA treatment causes Ca^2+^ hike and ER stress, leading to Calpain upregulation and activation. Upregulated calpain activity will eventually result in mitochondrial dysfunction, Caspases activation and neuronal apoptosis. Melatonin can effectively inhibit KA-induced calpain upregulation/activation and mitochondrial deterioration by alleviating Ca^2+^ overload and ER stress. Arrows indicate positive regulation, and blocks indicate negative regulation.

Previous studies indicated that excitotoxicity contributes to the neurodegenerative processes (Dong et al., 2009). Glutamate and related excitatory amino acids can induce neuronal apoptosis when administered both *in vivo* and *in vitro* (Reynolds and Hastings, 1995; Ding et al., 2015). In the present study, KA caused neuronal apoptosis and cytotoxicity in a dose-dependent manner (**Figure 1**). After KA treatment, we observed the release of Cyt C along with the activation of caspases, which act as terminal executors in the apoptosis pathway (Cohen, 1997). We observed the activation of caspase-12 and caspase-9, which participate in ER stress-mediated and mitochondria-mediated apoptosis pathways, respectively (**Figures 1, 2**) (Garcia de la Cadena and Massieu, 2016). These results suggest that ER stress and mitochondrial damages are both responsible for KA-induced neuronal apoptosis (**Figure 9**).

Calcium homeostasis is central to various cellular functions (Berridge et al., 2000). Calcium overload will impair cellular health, resulting in massive activation of proteases, phospholipases and cell death (Pinton et al., 2008). It was reported that the excessive accumulation of cytosolic Ca^2+^ can affect the scavenging roles of mitochondria and might lead to mitochondrial stress, including increases in mitochondrial ROS production and the collapse of mitochondrial membrane permeability (Mallilankaraman et al., 2012). ER is well-known to effectively buffer the cytosolic calcium concentration, mitochondria also participates in maintaining the cellular Ca^2+^ homeostasis (Pinton et al., 2008). Calcium transport from the ER to mitochondria plays a significant role in regulating cellular bioenergetics, ROS production and apoptosis induction. Mitochondria-associated ER-membranes (MAMs) may play a special role in the ER-mitochondria crosstalk (Marchi et al., 2014).

Recent report indicated that, in neurons, acute exposure to glutamate causes Parkin translocation to mitochondria in a calcium- and NMDA receptor-dependent manner. Parkin accumulates on MAMs following excitotoxicity, supporting a role of Parkin in ER-mitochondria crosstalk in mitochondrial homeostasis (Van Laar et al., 2015). Some MAMs proteins have been involved in mitochondrial dynamic fusion and fission including the mitofusin Mfn-2. Mfn-2 is enriched at the MAMs and its absence affects mitochondrial morphology and function (de Brito and Scorrano, 2008). Additionally, ER-located Mfn-2 is required for the connection with mitochondria by interacting directly with Mfn-1 or Mfn-2 on the mitochondria. The decrease in Mfn-2 could decrease Ca^2+^ traffic to the mitochondria (Guo et al., 2007). A very recent study showed that calpain activation in response to glutamate could result in post-translational protein degradation of Mfn-2, which is a novel mechanism regulating mitochondrial fusion during glutamate excitotoxicity (Wang et al., 2015). Similarly, we observed that Mfn-2 protein levels decreased while its mRNA level remained unchanged after KA treatment (**Figure 4**), suggesting that NMDA receptor activation by various ligands will result in the post-translational degradation of Mfn-2 and mitochondrial disorders in a like manner.

Kainic acid causes Ca^2+^ influx in N2a cells (**Figure 5**), which leads to activation of calpain and progressive neuronal death through mitochondria functional collapse (Harraz et al., 2012; Celso Constantino et al., 2014). Calpain belongs to the family of calcium-dependent non-lysosomal cysteine proteases, which is known to be involved in cytoskeleton and membrane attachments, signal transduction pathways, and apoptosis (McConkey and Orrenius, 1994; Chakraborti et al., 2012). In this study, the activity of calpain increased in response to KA-induced Ca^2+^ accumulation. To assay whether Ca^2+^ causes mitochondrial disorder through calpain activation, we used BAPTA-AM and calpeptin to inhibit the Ca^2+^ overloading and calpain activation. Both BAPTA-AM and calpeptin successfully inhibited the degradation of Mfn-2 and restored the normal amount and morphology of the mitochondria (**Figure 6**). These data further demonstrated that calpain activation by Ca^2+^ influx results in the post-translational degradation of Mfn-2 and mitochondrial impairments. Our study also suggests that ER stress plays a role in the mitochondrial disorder. ER stress inhibitor PBA decreased the expression of calpain and Ca^2+^ concentration. PBA also alleviated the degradation of Mfn-2 and inhibited the activation of caspase-3, restoring the function and morphology of mitochondria (**Figure 7**). These results reveal that mitochondrial dynamic impairment could be partially attributed to ER stress. All these observations suggest that the mitochondrial dynamic disorder was due to the aberrant degradation of Mfn-2 by calpain upregulation and activation, and that mitochondrial dysfunction could be the result of a defect in mitochondrial fusion (**Figure 3**).

Our study also suggested that ER stress also participates in KA-induced apoptosis directly. The activation of caspase-12 indicates the activation of the ER stress pathway (**Figures 1, 9**, caspase-12 pathway on the right side). As a proapoptotic unfolded protein response (UPR) factor, CHOP could also be activated by persistent ER stress (Malhi and Kaufman, 2011). KA treatment enhances the expression of GRP78 and CHOP, which could be activated by the UPR (Zheng et al., 2015). The simultaneous increases in expression of GRP78, CHOP and caspase-12 in the KA-treated group suggested that ER stress is involved in apoptosis directly (**Figure 7**).

Melatonin is a naturally occurring molecule with antioxidant properties (Hardeland, 2005; Reiter et al., 2009; Galano et al., 2011, 2013). Melatonin and its metabolites have the ability to scavenge ROS and reactive nitrogen species (RNS) (Galano et al., 2013). As a broad spectrum antioxidant (Zhang and Zhang, 2014; Manchester et al., 2015), melatonin has pleiotropic effects as well as neuroprotective properties (Negi et al., 2011; Miller et al., 2014). Melatonin acts against the elevation of lipid peroxidation induced by either KA or NMDA. Melatonin also blocked both KA- and NMDA-receptor mediated neuronal damage (Kim and Kwon, 1999). In addition, melatonin attenuates morphine-induced NMDA receptor subtype NR1 expression and decreases calcium concentration via modulating PKCγ activities in the spinal cord (Song et al., 2015). Accumulating evidence emphasizes contributions of melatonin toward the maintenance of ER and mitochondrial homeostasis, which made it an increasingly interesting pharmacological agent against neurodegenerative diseases (Hu et al., 2016; Uguz et al., 2016).

At the subcellular level, melatonin was found to attenuate methamphetamine-induced translocation of mitochondrial fission proteins, cytosolic calcium overload and cell death in SH-SY5Y cells (Parameyong et al., 2015). Melatonin can also suppress methamphetamine-triggered ER stress in C6 cells (Tungkum et al., 2017).

In our study, melatonin can effectively inhibit KA-induced calpain upregulation/activation and mitochondrial deterioration by alleviating Ca^2+^ overload and ER stress. Specifically, melatonin reduced the expression of c-caspase-9 and c-caspase-12 after KA treatment (**Figures 1F,G**), indicating the inhibitory effect on both mitochondrial dysfunction and ER stress related apoptosis pathways. Taken together, our study supported the hypothesis that melatonin has beneficial roles in countering neuronal death through blocking Ca^2+^ overload and ER stress.

## Author Contributions

FX and CS carried out cell culture, biochemical measurements and drafted the manuscript. They contributed equally to the work. WH carried out confocal imaging and the analysis of mitochondria dynamics. LX carried out ATP production assay. YW carried out calpain activity assay. ST participated in the preparation of manuscript. JZ participated in the measurement of LDH and cell viability. QC performed the statistical analysis. AS participated in the design and preparation of manuscript. JC conceived the study and participated in the coordination and preparation of manuscript. All authors read and approved the final draft.

## Conflict of Interest Statement

The authors declare that the research was conducted in the absence of any commercial or financial relationships that could be construed as a potential conflict of interest.

## Supplementary Material

FIGURE S1 **Melatonin ameliorates KA induced mitochondrial fragmentation in rat primary neurons. (A)** The morphology of mitochondria stained by Mito-tracker in KA and/or melatonin treated neurons. **(B)** Numbers of mitochondria in KA and/or melatonin treated neurons. **(C)** Average length of mitochondria in KA and/or melatonin treated neurons. ^∗^*P* < 0.05, ^∗∗^*P* < 0.01 vs. controls; ^#^*P* < 0.05, ^##^*P* < 0.01 vs. the KA group; significant difference from the respective values determined by one-way analysis of variance test, *n* = 3.

FIGURE S2 **Melatonin and PBA ameliorates KA induced Ca^2+^ elevation. (A)** Fura-2 AM probe was used to determine real-time ratio of F340/F380 in KA and/or melatonin-treated N2a cells. **(B**) Fura-2 AM probe was used to measure real-time ratio of F340/F380 in KA and/or PBA-treated N2a cells.

## References

[B1] Bahar E. Kim H. Yoon H. (2016). ER stress-mediated signaling: action potential and Ca(2+) as key players. *Int. J. Mol. Sci.* 17:E1558 10.3390/ijms17091558 PMC5037829 27649160

[B2] Berridge M. J. Lipp P. Bootman M. D. (2000). The versatility and universality of calcium signalling. *Nat. Rev. Mol. Cell Biol.* 1 11–21. 10.1038/35036035 11413485

[B3] Blatter L. A. Wier W. G. (1990). Intracellular diffusion, binding, and compartmentalization of the fluorescent calcium indicators indo-1 and fura-2. *Biophys. J.* 58 1491–1499. 10.1016/s0006-3495(90)82494-2 2275965 PMC1281101

[B4] Bleakman D. Lodge D. (1998). Neuropharmacology of AMPA and kainate receptors. *Neuropharmacology* 37 1187–1204. 10.1016/S0028-3908(98)00139-7 9849657

[B5] Cajochen C. Kräuchi K. Wirz-Justice A. (2003). Role of melatonin in the regulation of human circadian rhythms and sleep. *J. Neuroendocrinol.* 15 432–437. 10.1046/j.1365-2826.2003.00989.x 12622846

[B6] Celso Constantino L. Tasca C. I. Boeck C. R. (2014). The role of NMDA receptors in the development of brain resistance through pre- and postconditioning. *Aging Dis.* 5 430–441. 10.14336/ad.2014.0500430 25489494 PMC4249812

[B7] Chakraborti S. Alam M. N. Paik D. Shaikh S. Chakraborti T. (2012). Implications of calpains in health and diseases. *Indian J. Biochem. Biophys.* 49 316–328. 23259318

[B8] Chen H. Chan D. C. (2009). Mitochondrial dynamics–fusion, fission, movement, and mitophagy–in neurodegenerative diseases. *Hum. Mol. Genet.* 18 R169–R176. 10.1093/hmg/ddp326 19808793 PMC2758711

[B9] Chen M. Guerrero A. D. Huang L. Shabier Z. Pan M. Tan T. H. (2007). Caspase-9-induced mitochondrial disruption through cleavage of anti-apoptotic BCL-2 family members. *J. Biol. Chem.* 282 33888–33895. 10.1074/jbc.M702969200 17893147

[B10] China Ministry of Health Publication (2001). http://www.nsfc.gov.cn/nsfc/cen/pfzl/pufanew/20110801_11.htm

[B11] Cohen G. M. (1997). Caspases: the executioners of apoptosis. *Biochem. J.* 326(Pt 1) 1–16. 10.1042/bj3260001 9337844 PMC1218630

[B12] Crespo-Biel N. Camins A. Canudas A. M. Pallas M. (2010). Kainate-induced toxicity in the hippocampus: potential role of lithium. *Bipolar Disord.* 12 425–436. 10.1111/j.1399-5618.2010.00825.x 20636640

[B13] Crespo-Biel N. Canudas A. M. Camins A. Pallas M. (2007). Kainate induces AKT, ERK and cdk5/GSK3beta pathway deregulation, phosphorylates tau protein in mouse hippocampus. *Neurochem. Int.* 50 435–442. 10.1016/j.neuint.2006.10.002 17116346

[B14] de Brito O. M. Scorrano L. (2008). Mitofusin 2 tethers endoplasmic reticulum to mitochondria. *Nature* 456 605–610. 10.1038/nature07534 19052620

[B15] De Vos K. J. Sheetz M. P. (2007). Visualization and quantification of mitochondrial dynamics in living animal cells. *Methods Cell Biol.* 80 627–682. 10.1016/S0091-679X(06)80030-0 17445716

[B16] Ding Z.-J. Chen X. I. N. Tang X.-X. Wang X. I. Song Y.-L. Chen X.-D. (2015). Apoptosis-inducing factor and calpain upregulation in glutamate-induced injury of rat spiral ganglion neurons. *Mol. Med. Rep.* 12 1685–1692. 10.3892/mmr.2015.3626 25891494 PMC4464299

[B17] Dong X. X. Wang Y. Qin Z. H. (2009). Molecular mechanisms of excitotoxicity and their relevance to pathogenesis of neurodegenerative diseases. *Acta Pharmacol. Sin.* 30 379–387. 10.1038/aps.2009.24 19343058 PMC4002277

[B18] Galano A. Tan D. X. Reiter R. J. (2011). Melatonin as a natural ally against oxidative stress: a physicochemical examination. *J. Pineal Res.* 51 1–16. 10.1111/j.1600-079X.2011.00916.x 21752095

[B19] Galano A. Tan D. X. Reiter R. J. (2013). On the free radical scavenging activities of melatonin’s metabolites, AFMK and AMK. *J. Pineal Res.* 54 245–257. 10.1111/jpi.12010 22998574

[B20] Garcia de la Cadena S. Massieu L. (2016). Caspases and their role in inflammation and ischemic neuronal death. Focus on caspase-12. *Apoptosis* 21 763–777. 10.1007/s10495-016-1247-0 27142195

[B21] Golstein P. Kroemer G. (2007). Cell death by necrosis: towards a molecular definition. *Trends Biochem. Sci.* 32 37–43. 10.1016/j.tibs.2006.11.001 17141506

[B22] Gouriou Y. Bijlenga P. Demaurex N. (2013). Mitochondrial Ca2+ uptake from plasma membrane Cav3.2 protein channels contributes to ischemic toxicity in PC12 cells. *J. Biol. Chem.* 288 12459–12468. 10.1074/jbc.M112.428128 23508951 PMC3642294

[B23] Greene N. P. Lee D. E. Brown J. L. Rosa M. E. Brown L. A. Perry R. A. (2015). Mitochondrial quality control, promoted by PGC-1α, is dysregulated by Western diet-induced obesity and partially restored by moderate physical activity in mice. *Physiol. Rep.* 3:e12470 10.14814/phy2.12470 PMC4552545 26177961

[B24] Guo X. Chen K. H. Guo Y. Liao H. Tang J. Xiao R. P. (2007). Mitofusin 2 triggers vascular smooth muscle cell apoptosis via mitochondrial death pathway. *Circ. Res.* 101 1113–1122. 10.1161/CIRCRESAHA.107.157644 17901359

[B25] Halestrap A. P. (2009). What is the mitochondrial permeability transition pore? *J. Mol. Cell Cardiol.* 46 821–831. 10.1016/j.yjmcc.2009.02.021 19265700

[B26] Hardeland R. (2005). Antioxidative protection by melatonin: multiplicity of mechanisms from radical detoxification to radical avoidance. *Endocrine* 27 119–130. 10.1385/ENDO:27:2:119 16217125

[B27] Harraz M. M. Eacker S. M. Wang X. Dawson T. M. Dawson V. L. (2012). MicroRNA-223 is neuroprotective by targeting glutamate receptors. *Proc. Natl. Acad. Sci. U.S.A.* 109 18962–18967. 10.1073/pnas.1121288109 23112146 PMC3503176

[B28] Hu W. Ma Z. Di S. Jiang S. Li Y. Fan C. (2016). Snapshot: implications for melatonin in endoplasmic reticulum homeostasis. *Br. J. Pharmacol.* 173 3431–3442. 10.1111/bph.13651 27759160 PMC5120159

[B29] Hu X. Li P. Guo Y. Wang H. Leak R. K. Chen S. (2012). Microglia/macrophage polarization dynamics reveal novel mechanism of injury expansion after focal cerebral ischemia. *Stroke* 43 3063–3070. 10.1161/STROKEAHA.112.659656 22933588

[B30] Isaev N. K. Stelmashook E. V. Dirnagl U. Andreeva N. A. Manuhova L. Vorobjev V. S. (2002). Neuroprotective effects of the antifungal drug clotrimazole. *Neuroscience* 113 47–53. 10.1016/S0306-4522(02)00164-1 12123683

[B31] Ishihara N. Fujita Y. Oka T. Mihara K. (2006). Regulation of mitochondrial morphology through proteolytic cleavage of OPA1. *EMBO J.* 25 2966–2977. 10.1038/sj.emboj.7601184 16778770 PMC1500981

[B32] Jain A. Sharma D. Suhalka P. Sukhwal P. Bhatnagar M. (2013). Changes in the density of nitrergic neurons in the hippocampus of rats following kainic acid and melatonin administration. *Physiol. Res.* 62 197–203. 23234414 10.33549/physiolres.932295

[B33] Jean-Louis G. von Gizycki H. Zizi F. (1998). Melatonin effects on sleep, mood, and cognition in elderly with mild cognitive impairment. *J. Pineal Res.* 25 177–183. 10.1111/j.1600-079X.1998.tb00557.x 9745987

[B34] Johri A. Beal M. F. (2012). Mitochondrial dysfunction in neurodegenerative diseases. *J. Pharmacol. Exp. Ther.* 342 619–630. 10.1124/jpet.112.192138 22700435 PMC3422529

[B35] Kim H. Lee J. Y. Park K. J. Kim W. H. Roh G. S. (2016). A mitochondrial division inhibitor, Mdivi-1, inhibits mitochondrial fragmentation and attenuates kainic acid-induced hippocampal cell death. *BMC Neurosci.* 17:33 10.1186/s12868-016-0270-y PMC4902937 27287829

[B36] Kim H. J. Kwon J. S. (1999). Effects of placing micro-implants of melatonin in striatum on oxidative stress and neuronal damage mediated by N-methyl-D-aspartate (n.d.) and non-NMDA receptors. *Arch. Pharm. Res.* 22 35–43. 10.1007/BF02976433 10071957

[B37] Kruman I. Guo Q. Mattson M. P. (1998). Calcium and reactive oxygen species mediate staurosporine-induced mitochondrial dysfunction and apoptosis in PC12 cells. *J. Neurosci. Res.* 51 293–308. 10.1002/(SICI)1097-4547(19980201)51:3<293::AID-JNR3>3.0.CO;2-B 9486765

[B38] Lai T. W. Zhang S. Wang Y. T. (2014). Excitotoxicity and stroke: identifying novel targets for neuroprotection. *Prog. Neurobiol.* 115 157–188. 10.1016/j.pneurobio.2013.11.006 24361499

[B39] Li H. Wang Y. Feng D. Liu Y. Xu M. Gao A. (2014). Alterations in the time course of expression of the Nox family in the brain in a rat experimental cerebral ischemia and reperfusion model: effects of melatonin. *J. Pineal Res.* 57 110–119. 10.1111/jpi.12148 24867613

[B40] Li S. Y. Jia Y. H. Sun W. G. Tang Y. An G. S. Ni J. H. (2010). Stabilization of mitochondrial function by tetramethylpyrazine protects against kainate-induced oxidative lesions in the rat hippocampus. *Free Radic. Biol. Med.* 48 597–608. 10.1016/j.freeradbiomed.2009.12.004 20006702

[B41] Lindholm D. Wootz H. Korhonen L. (2006). ER stress and neurodegenerative diseases. *Cell Death Differ.* 13 385–392. 10.1038/sj.cdd.4401778 16397584

[B42] Liu B. Yuan B. Zhang L. Mu W. Wang C. (2015). ROS/p38/p53/Puma signaling pathway is involved in emodin-induced apoptosis of human colorectal cancer cells. *Int. J. Clin. Exp. Med.* 8 15413–15422. 26629030 PMC4658919

[B43] Logue S. E. Cleary P. Saveljeva S. Samali A. (2013). New directions in ER stress-induced cell death. *Apoptosis* 18 537–546. 10.1007/s10495-013-0818-6 23430059

[B44] Malhi H. Kaufman R. J. (2011). Endoplasmic reticulum stress in liver disease. *J. Hepatol.* 54 795–809. 10.1016/j.jhep.2010.11.005 21145844 PMC3375108

[B45] Mallilankaraman K. Cárdenas C. Doonan P. Chandramoorthy H. C. Irrinki K. M. Golenár T. (2012). MCUR1 is an essential component of mitochondrial Ca(2+) uptake that regulates cellular metabolism. *Nat. Cell Biol.* 14 1336–1343. 10.1038/ncb2622 23178883 PMC3511605

[B46] Manchester L. C. Coto-Montes A. Boga J. A. Andersen L. P. H. Zhou Z. Galano A. (2015). Melatonin: an ancient molecule that makes oxygen metabolically tolerable. *J. Pineal Res.* 59 403–419. 10.1111/jpi.12267 26272235

[B47] Manev H. Favaron M. Guidotti A. Costa E. (1989). Delayed increase of Ca2+ influx elicited by glutamate: role in neuronal death. *Mol. Pharmacol.* 36 106–112. 2568579

[B48] Marchi S. Patergnani S. Pinton P. (2014). The endoplasmic reticulum-mitochondria connection: one touch, multiple functions. *Biochim. Biophys. Acta* 1837 461–469. 10.1016/j.bbabio.2013.10.015 24211533

[B49] McConkey D. J. Orrenius S. (1994). Signal transduction pathways to apoptosis. *Trends Cell Biol.* 4 370–375. 10.1016/0962-8924(94)90087-6 14731626

[B50] McGeer E. G. McGeer P. L. (1978). Some factors influencing the neurotoxicity of intrastriatal injections of kainic acid. *Neurochem. Res.* 3 501–517. 10.1007/BF00966331 34114

[B51] Mesenge C. Margaill I. Verrecchia C. Allix M. Boulu R. G. Plotkine M. (1998). Protective effect of melatonin in a model of traumatic brain injury in mice. *J. Pineal Res.* 25 41–46. 10.1111/j.1600-079X.1998.tb00384.x 9694403

[B52] Milatovic D. Zivin M. Gupta R. C. Dettbarn W. D. (2001). Alterations in cytochrome c oxidase activity and energy metabolites in response to kainic acid-induced status epilepticus. *Brain Res.* 912 67–78. 10.1016/S0006-8993(01)02657-9 11520494

[B53] Miller S. L. Yawno T. Alers N. O. Castillo-Melendez M. Supramaniam V. G. VanZyl N. (2014). Antenatal antioxidant treatment with melatonin to decrease newborn neurodevelopmental deficits and brain injury caused by fetal growth restriction. *J. Pineal Res.* 56 283–294. 10.1111/jpi.12121 24456220

[B54] Nasca A. Legati A. Baruffini E. Nolli C. Moroni I. Ardissone A. (2016). Biallelic mutations in DNM1L are associated with a slowly progressive infantile encephalopathy. *Hum. Mutat.* 37 898–903. 10.1002/humu.23033 27328748 PMC5108486

[B55] Negi G. Kumar A. Sharma S. S. (2011). Melatonin modulates neuroinflammation and oxidative stress in experimental diabetic neuropathy: effects on NF-kappaB and Nrf2 cascades. *J. Pineal Res.* 50 124–131. 10.1111/j.1600-079X.2010.00821.x 21062351

[B56] Nicholls D. G. (2004). Mitochondrial dysfunction and glutamate excitotoxicity studied in primary neuronal cultures. *Curr. Mol. Med.* 4 149–177. 10.2174/1566524043479239 15032711

[B57] Osada N. Kosuge Y. Ishige K. Ito Y. (2010). Characterization of neuronal and astroglial responses to ER stress in the hippocampal CA1 area in mice following transient forebrain ischemia. *Neurochem. Int.* 57 1–7. 10.1016/j.neuint.2010.03.017 20362024

[B58] Parameyong A. Govitrapong P. Chetsawang B. (2015). Melatonin attenuates the mitochondrial translocation of mitochondrial fission proteins and Bax, cytosolic calcium overload and cell death in methamphetamine-induced toxicity in neuroblastoma SH-SY5Y cells. *Mitochondrion* 24 1–8. 10.1016/j.mito.2015.07.004 26176977

[B59] Park S.-Y. Jang W.-J. Yi E.-Y. Jang J.-Y. Jung Y. Jeong J.-W. (2010). Melatonin suppresses tumor angiogenesis by inhibiting HIF-1α stabilization under hypoxia. *J. Pineal Res.* 48 178–184. 10.1111/j.1600-079X.2009.00742.x 20449875

[B60] Pinton P. Giorgi C. Siviero R. Zecchini E. Rizzuto R. (2008). Calcium and apoptosis: ER-mitochondria Ca2+ transfer in the control of apoptosis. *Oncogene* 27 6407–6418. 10.1038/onc.2008.308 18955969 PMC2844952

[B61] Prieto-Domínguez N. Ordóñez R. Fernández A. Méndez-Blanco C. Baulies A. Garcia-Ruiz C. (2016). Melatonin-induced increase in sensitivity of human hepatocellular carcinoma cells to sorafenib is associated with reactive oxygen species production and mitophagy. *J. Pineal Res.* 61 396–407. 10.1111/jpi.12358 27484637 PMC5018464

[B62] Reiter R. J. Paredes S. D. Manchester L. C. Tan D. X. (2009). Reducing oxidative/nitrosative stress: a newly-discovered genre for melatonin. *Crit. Rev. Biochem. Mol. Biol.* 44 175–200. 10.1080/10409230903044914 19635037

[B63] Reynolds I. J. Hastings T. G. (1995). Glutamate induces the production of reactive oxygen species in cultured forebrain neurons following NMDA receptor activation. *J. Neurosci.* 15(5 Pt 1) 3318–3327. 7751912 10.1523/JNEUROSCI.15-05-03318.1995 PMC6578215

[B64] Sano R. Reed J. C. (2013). ER stress-induced cell death mechanisms. *Biochim. Biophys. Acta* 1833 3460–3470. 10.1016/j.bbamcr.2013.06.028 23850759 PMC3834229

[B65] Schinder A. F. Olson E. C. Spitzer N. C. Montal M. (1996). Mitochondrial dysfunction is a primary event in glutamate neurotoxicity. *J. Neurosci.* 16 6125–6133. 8815895 10.1523/JNEUROSCI.16-19-06125.1996 PMC6579180

[B66] Shen Q. Yamano K. Head B. P. Kawajiri S. Cheung J. T. Wang C. (2014). Mutations in Fis1 disrupt orderly disposal of defective mitochondria. *Mol. Biol. Cell* 25 145–159. 10.1091/mbc.E13-09-0525 24196833 PMC3873885

[B67] Song L. Wu C. Zuo Y. (2015). Melatonin prevents morphine-induced hyperalgesia and tolerance in rats: role of protein kinase C and N-methyl-D-aspartate receptors. *BMC Anesthesiol.* 15:12 10.1186/1471-2253-15-12 PMC4350305 25745356

[B68] Sperk G. Lassmann H. Baran H. Kish S. J. Seitelberger F. Hornykiewicz O. (1983). Kainic acid induced seizures: neurochemical and histopathological changes. *Neuroscience* 10 1301–1315. 10.1016/0306-4522(83)90113-6 6141539

[B69] Tombal B. Denmeade S. R. Isaacs J. T. (1999). Assessment and validation of a microinjection method for kinetic analysis of [Ca2+]i in individual cells undergoing apoptosis. *Cell Calcium* 25 19–28. 10.1054/ceca.1998.0005 10191957

[B70] Tungkum W. Jumnongprakhon P. Tocharus C. Govitrapong P. Tocharus J. (2017). Melatonin suppresses methamphetamine-triggered endoplasmic reticulum stress in C6 cells glioma cell lines. *J. Toxicol. Sci.* 42 63–71. 10.2131/jts.42.63 28070110

[B71] Uguz A. C. Demirci K. Espino J. (2016). The importance of melatonin and mitochondria interaction in mood disorders and schizophrenia: a current assessment. *Curr. Med. Chem.* 23 2146–2158. 10.2174/0929867323666160428105849 27121187

[B72] Van Laar V. S. Roy N. Liu A. Rajprohat S. Arnold B. Dukes A. A. (2015). Glutamate excitotoxicity in neurons triggers mitochondrial and endoplasmic reticulum accumulation of Parkin, and, in the presence of N-acetyl cysteine, mitophagy. *Neurobiol. Dis.* 74 180–193. 10.1016/j.nbd.2014.11.015 25478815 PMC4322770

[B73] Wang Q. Yu S. Simonyi A. Sun G. Y. Sun A. Y. (2005). Kainic acid-mediated excitotoxicity as a model for neurodegeneration. *Mol. Neurobiol.* 31 3–16. 10.1385/MN:31:1-3:003 15953808

[B74] Wang W. Zhang F. Li L. Tang F. Siedlak S. L. Fujioka H. (2015). MFN2 couples glutamate excitotoxicity and mitochondrial dysfunction in motor neurons. *J. Biol. Chem.* 290 168–182. 10.1074/jbc.M114.617167 25416777 PMC4281719

[B75] Wang Y. Han R. Liang Z. Q. Wu J. C. Zhang X. D. Gu Z. L. (2008). An autophagic mechanism is involved in apoptotic death of rat striatal neurons induced by the non-N-methyl-D-aspartate receptor agonist kainic acid. *Autophagy* 4 214–226. 10.4161/auto.5369 18094625

[B76] Wang Y. Qin Z. H. (2010). Molecular and cellular mechanisms of excitotoxic neuronal death. *Apoptosis* 15 1382–1402. 10.1007/s10495-010-0481-0 20213199

[B77] Wrogemann K. Pena S. D. (1976). Mitochondrial calcium overload: a general mechanism for cell-necrosis in muscle diseases. *Lancet* 1 672–674. 10.1016/S0140-6736(76)92781-1 73643

[B78] Wu H. Y. Lynch D. R. (2006). Calpain and synaptic function. *Mol. Neurobiol.* 33 215–236. 10.1385/MN:33:3:215 16954597

[B79] Wu Y.-H. Swaab D. F. (2005). The human pineal gland and melatonin in aging and Alzheimer’s disease. *J. Pineal Res.* 38 145–152. 10.1111/j.1600-079X.2004.00196.x 15725334

[B80] Ye J. Han Y. Chen X. Xie J. Liu X. Qiao S. (2014). L-carnitine attenuates H2O2-induced neuron apoptosis via inhibition of endoplasmic reticulum stress. *Neurochem. Int.* 78 86–95. 10.1016/j.neuint.2014.08.009 25220073

[B81] Yildiz-Unal A. Korulu S. Karabay A. (2015). Neuroprotective strategies against calpain-mediated neurodegeneration. *Neuropsychiatr. Dis. Treat.* 11 297–310. 10.2147/NDT.S78226 25709452 PMC4327398

[B82] Zhang H. M. Zhang Y. (2014). Melatonin: a well-documented antioxidant with conditional pro-oxidant actions. *J. Pineal Res.* 57 131–146. 10.1111/jpi.12162 25060102

[B83] Zhang X. M. Zhu J. (2011). Kainic Acid-induced neurotoxicity: targeting glial responses and glia-derived cytokines. *Curr. Neuropharmacol.* 9 388–398. 10.2174/157015911795596540 22131947 PMC3131729

[B84] Zhao J. Lendahl U. Nistér M. (2013). Regulation of mitochondrial dynamics: convergences and divergences between yeast and vertebrates. *Cell. Mol. Life Sci.* 70 951–976. 10.1007/s00018-012-1066-6 22806564 PMC3578726

[B85] Zheng G. F. Cai Z. Meng X. K. Zhang Y. Zhu W. Pang X. Y. (2015). Unfolded protein response mediated JNK/AP-1 signal transduction, a target for ovarian cancer treatment. *Int. J. Clin. Exp. Pathol.* 8 6505–6511. 26261528 PMC4525862

