# Supplementary figures and images for: Melatonin Mediates Protective Effects against Kainic Acid-Induced Neuronal Death through Safeguarding ER Stress and Mitochondrial Disturbance
Source: Front Mol Neurosci. 2017 Feb 28;10:49. doi: 10.3389/fnmol.2017.00049 (PMC5329003; doi:10.3389/fnmol.2017.00049)

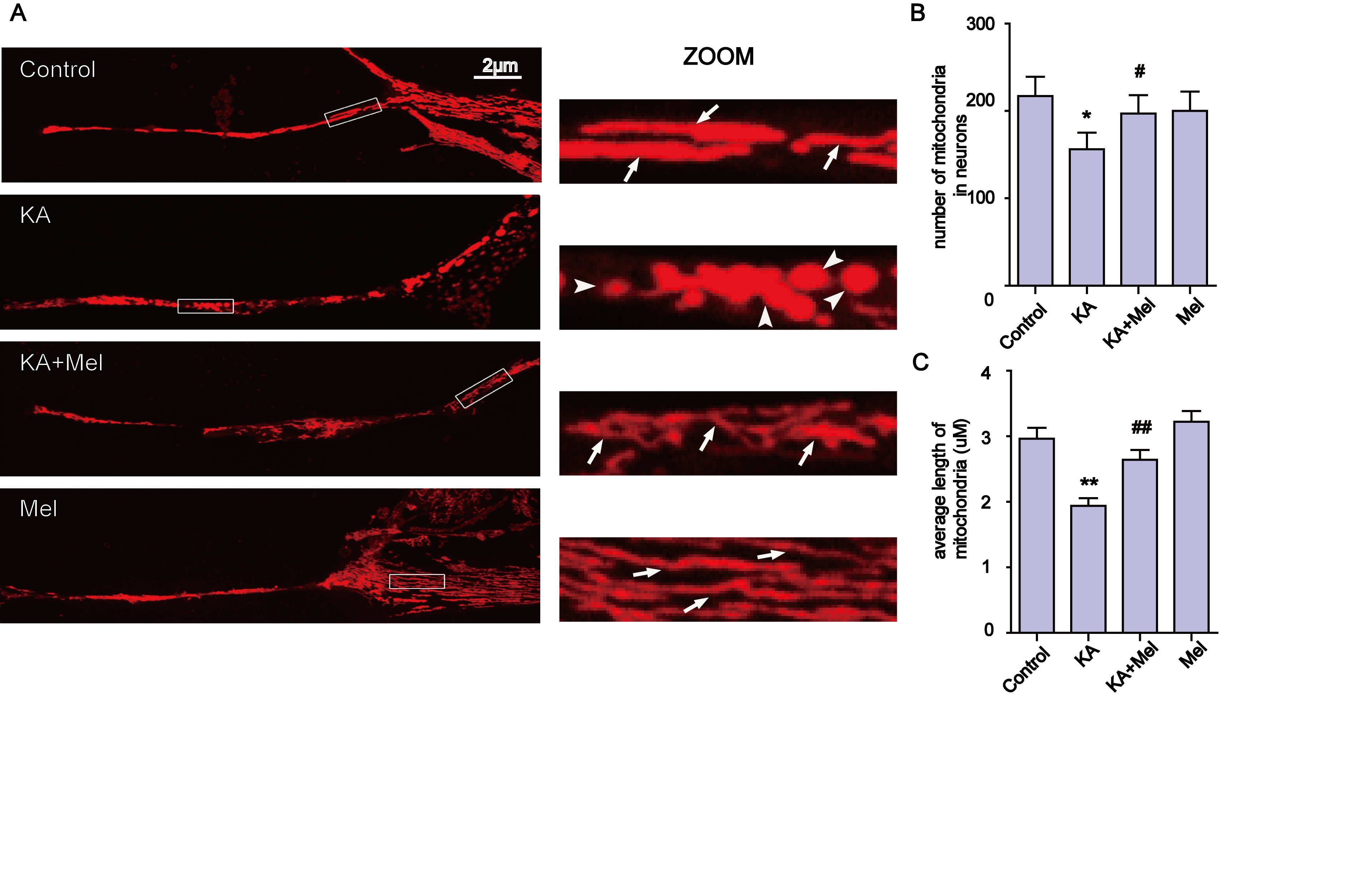

Supplement: FIGURE S1 — Melatonin ameliorates KA induced mitochondrial fragmentation in rat primary neurons. (A) The morphology of mitochondria stained by Mito-tracker in KA and/or melatonin treated neurons. (B) Numbers of mitochondria in KA and/or melatonin treated neurons. (C) Average length of mitochondria in KA and/or melatonin treated neurons. ∗P < 0.05, ∗∗P < 0.01 vs. controls; #P < 0.05, ##P < 0.01 vs. the KA group; significant difference from the respective values determined by one-way analysis of variance test, n = 3. [file Image_1.tif]

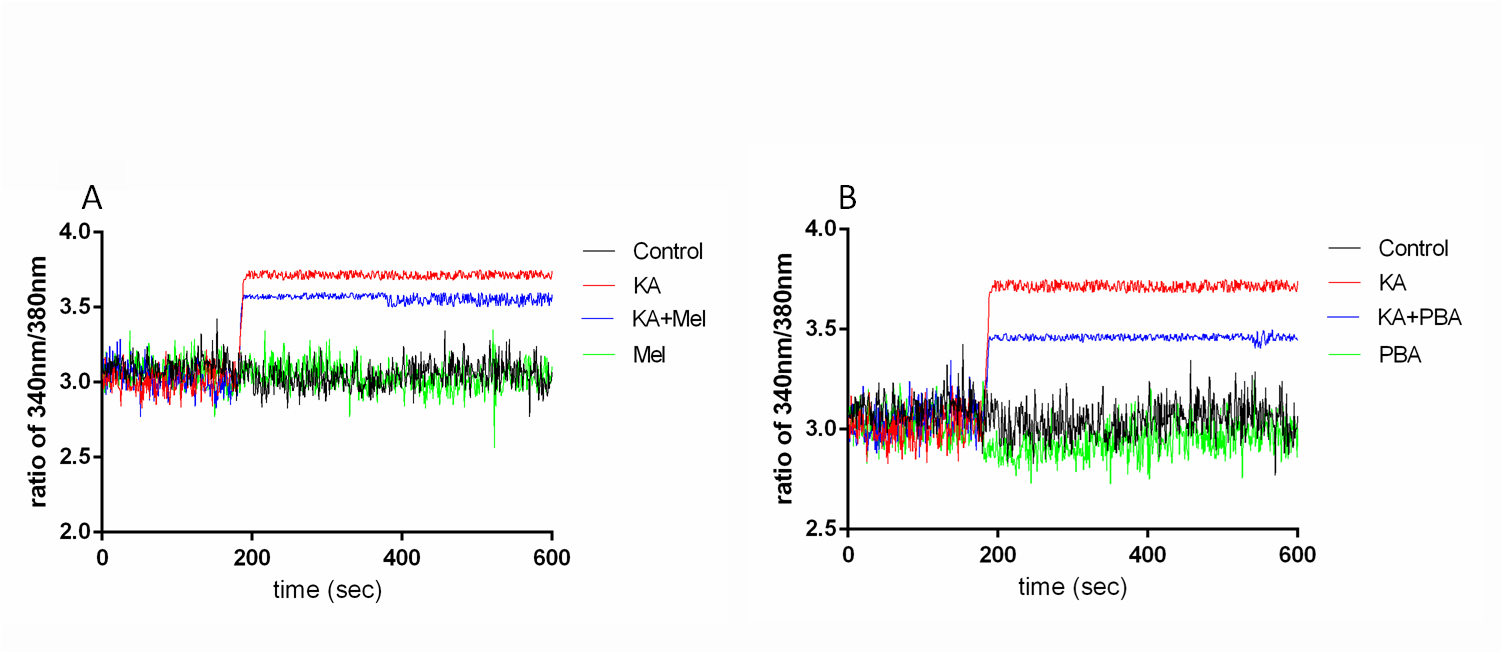

Supplement: FIGURE S2 — Melatonin and PBA ameliorates KA induced Ca2+ elevation. (A) Fura-2 AM probe was used to determine real-time ratio of F340/F380 in KA and/or melatonin-treated N2a cells. (B) Fura-2 AM probe was used to measure real-time ratio of F340/F380 in KA and/or PBA-treated N2a cells. [file Image_2.tif]
